# From pluripotency to forebrain patterning: an in vitro journey astride embryonic stem cells

**DOI:** 10.1007/s00018-014-1596-1

**Published:** 2014-03-19

**Authors:** Giuseppe Lupo, Michele Bertacchi, Nicoletta Carucci, Gabriella Augusti-Tocco, Stefano Biagioni, Federico Cremisi

**Affiliations:** 1 Department of Biology and Biotechnology “C. Darwin”, Sapienza University of Rome, Piazzale Aldo Moro 5, 00185 Rome, Italy; 2 Istituto Pasteur-Fondazione Cenci Bolognetti, Sapienza University of Rome, Piazzale Aldo Moro 5, 00185 Rome, Italy; 3 Scuola Normale Superiore di Pisa, Piazza dei Cavalieri 7, 56100 Pisa, Italy

**Keywords:** Epiblast, Neural induction, Neuroectoderm, Anteroposterior patterning, Telencephalon, Eye field

## Abstract

Embryonic stem cells (ESCs) have been used extensively as in vitro models of neural development and disease, with special efforts towards their conversion into forebrain progenitors and neurons. The forebrain is the most complex brain region, giving rise to several fundamental structures, such as the cerebral cortex, the hypothalamus, and the retina. Due to the multiplicity of signaling pathways playing different roles at distinct times of embryonic development, the specification and patterning of forebrain has been difficult to study in vivo. Research performed on ESCs in vitro has provided a large body of evidence to complement work in model organisms, but these studies have often been focused more on cell type production than on cell fate regulation. In this review, we systematically reassess the current literature in the field of forebrain development in mouse and human ESCs with a focus on the molecular mechanisms of early cell fate decisions, taking into consideration the specific culture conditions, exogenous and endogenous molecular cues as described in the original studies. The resulting model of early forebrain induction and patterning provides a useful framework for further studies aimed at reconstructing forebrain development in vitro for basic research or therapy.

## Introduction

The forebrain is the most complex region of the vertebrate central nervous system (CNS), containing several elaborate neural centers, such as the cerebral cortex, the basal ganglia, the thalamus, the hypothalamus, and the retina.

This anatomical complexity is achieved through sequential inductive interactions, progressively restricting the fate of pluripotent embryonic cells towards specific neural identities. The first step in forebrain development is neural induction, causing neuroectoderm formation in the dorsal region of the ectoderm germ layer [1–3]. This is accompanied and/or followed by anteroposterior (AP) regionalization of the neuroectoderm into four main domains: the presumptive forebrain, midbrain, hindbrain, and spinal cord [4]. Local patterning mechanisms within the forebrain further subdivide it into the primordia of the telencephalon, of the retina (or eye field), of the hypothalamus and of the diencephalon [5, 6]. Patterning along the dorsoventral (DV) axis parcels each of these areas into progenitor domains committed to form specific structures, such as the pallium and the basal ganglia in the telencephalon [7], and the retina and the optic stalk in the eye field [8].

The molecular mechanisms controlling these events have been studied extensively in vertebrate model organisms and especially fish, frog, chick, and mouse embryos. While studies in these systems have identified the main genetic pathways controlling early neural development, they have not entirely reconstructed the molecular events underlying forebrain regionalization. The reason for this is that, in whole organisms, it is difficult to separate the roles of multiple molecular pathways acting simultaneously and to manipulate specific signaling events without interfering with earlier or later roles for the same pathways.

ESCs are pluripotent cells derived from pre-implantation mammalian embryos, which share many similarities with progenitors found in early embryos before gastrulation [9–11]. Mouse ESCs (mESCs) and human ESCs (hESCs) are powerful tools for the study of forebrain development in vitro. Chemically defined culture conditions have been established, allowing in depth investigation of the molecular signals controlling cell fate specification. hESCs have provided a unique experimental system to study the genetic control of embryogenesis in human cells [12]. The development of induced pluripotent stem cells from human patients has opened new avenues to translate developmental studies in hESCs into therapeutic applications [13]. The main inductive signals controlling forebrain specification are conserved in model organisms and in ESCs, thus confirming that ESCs are apt to model early forebrain development in vitro [14, 15]. Nonetheless, significant differences exist in the techniques used for ESC culture and neuralization, including culture media composition, the substrates used for adherent culture, and the choice between adherent or floating aggregate culture. There is still a poor understanding of how different culture conditions can affect cell-fate choices. Moreover, the complexity of the signals endogenously produced by differentiating ESCs and their impact on the specification of distinct neural identities are not yet fully characterized.

Here, we review the journey of ESCs from pluripotency to specialized forebrain cells in a culture dish. We discuss how the specification of different forebrain fates in ESCs recapitulates the sequence of events known to take place in vivo, but we also highlight new insights obtained thanks to the use of ESCs. Furthermore, we discuss some of the challenges lying ahead in this field and especially the importance of fully characterizing the influence of the chosen culture conditions on neural specification and patterning of ESCs.

## Neuralization of ESCs: from pluripotent stem cells to neural progenitors

Vertebrate neural induction was first discovered in amphibians, where the dorsal mesendoderm of gastrulating embryos induces neuroectoderm formation in the dorsal ectoderm, diverting it from epidermal differentiation [16]. Similar neural inducing centers, collectively designated as the gastrula organizer, exist in embryos across vertebrates, such as the shield in fish, Hensen’s node in chick and the node in mice [1, 2, 17].

### Transforming growth factor β (TGFβ) signals inhibit neural conversion of ESCs

Frog embryos have been pivotal in elucidating the molecular signals produced by the organizer. Studies performed in this system have recently been reviewed elsewhere (e.g., [1, 16]). Essentially, they established that two branches of the TGFβ signaling pathway, namely Activin/Nodal and bone morphogenetic protein (BMP) signaling, strongly inhibit neural development. Their activity needs to be repressed in the ectoderm by organizer signals for neural induction to occur, while elevated TGFβ signaling drives ectodermal cells towards non-neural fates (such as epidermis and mesendoderm). This mechanism of neural induction was initially uncovered by in vitro assays in ectodermal explants [1, 16], but it has subsequently been validated by genetic studies in vivo in frog and fish embryos [18–21].

Genetic manipulation of TGFβ signaling in mice has shown that Activin/Nodal and BMP pathways are essential for early developmental steps preceding neural induction [22]. For that reason, as well as the redundancy of TGFβ ligands and their antagonists in neural induction [18–21, 23], analyzing the roles of these pathways in mammalian neural development in vivo has proven difficult. ESC cultures have provided an alternative system to confirm in mammals the critical role of TGFβ signaling in neuroectoderm formation.

mESCs and hESCs are pluripotent cells derived from the inner cell mass (ICM) of blastocyst stage embryos [9–12]. In both cases, an autoregulatory network involving the transcription factors Oct4, Nanog, and Sox2 plays key roles in the maintenance of pluripotency and suppression of lineage-specific genes, including neural determination genes [24]. Studies performed in mESCs and hESCs support the notion that TGFβ signaling opposes neural fate specification and that pluripotent progenitors undertake a neural developmental pathway when they are shielded from this anti-neuralizing influence. Crucially, ESCs have been instrumental in understanding that the ability of Activin/Nodal and BMP pathways to repress neural development is linked to their roles in maintaining ESC pluripotency and self-renewal (see below).

Unexpectedly, mESCs and hESCs show different reliance on TGFβ signaling for the maintenance of pluripotency. The molecular mechanisms of pluripotency have been reviewed elsewhere (e.g., [9–11, 24]). Briefly, in mESCs, BMP signaling cooperates with the LIF pathway in the maintenance of Oct4–Nanog–Sox2 expression and pluripotency [25]. Unexpectedly, BMP and LIF signaling are ineffective in supporting hESC pluripotency, which requires the collaborative actions of Activin/Nodal and fibroblast growth factor (FGF) pathways [26]. This apparent paradox has been at least partially solved thanks to the isolation of pluripotent cells from the epiblast of pre-gastrula stage mouse embryos, which have been named epiblast stem cells (EpiSCs) [27, 28]. EpiSCs share a number of features with hESCs, but not mESCs, including reliance on Activin/Nodal and FGF signaling, rather than BMP and LIF, for their pluripotency. mESCs and EpiSCs can be converted into each other [9–11], suggesting that pluripotency is a dynamic, developmentally regulated process, which is supported by different extrinsic cues at different developmental stages. These observations have led to the idea that hESCs represent a developmental stage closer to epiblast progenitors (and hence EpiSCs) than to ICM progenitors (and mESCs) [29].

BMP and Activin/Nodal signaling play corresponding roles in supporting pluripotency and repressing neural specification in mESCs and hESCs, respectively. In mESCs, LIF is insufficient to block neural differentiation in serum free cultures [25]. By activating the Smad1/5/8 pathway, BMPs, which are present in serum, induce expression of *Id* genes that are necessary, and sufficient together with LIF, to allow self-renewal [25]. Activation of the BMP pathway alone is not sufficient to maintain pluripotency. Upon LIF withdrawal, Id-expressing mESCs differentiate, but do not give rise to neural lineages [25]. Thus, the blockade of neural-specific transcription factors by Id proteins enables the self-renewal response of mESCs to LIF signaling. In hESCs, Smad2/3 work downstream of Activin/Nodal signaling to directly bind and promote expression of the *Nanog* gene [30, 31]. Nanog in turn acts as a strong repressor of neuroectoderm specification [31]. Thus, the anti-neuralizing activities of TGFβ signaling and pluripotent stem cell self-renewal appear to be closely interconnected at the level of the pluripotency core regulatory network. In agreement with studies in ESCs, analysis of mouse mutants for BMP receptor or *Nodal* genes showed that TGFβ signaling prevents premature neural induction in the developing epiblast in vivo [32, 33]. In mESCs and/or hESCs, besides their respective roles in pluripotency, Activin/Nodal signaling has been associated with primitive endoderm and mesendoderm differentiation [34–36], while BMP signaling can promote, depending on the context, trophectoderm [37, 38], primitive endoderm [38, 39], mesendoderm [38, 40], or non-neural ectoderm differentiation [41, 42]. Thus, TGFβ signaling can negate neuroectoderm formation in ESCs both by supporting pluripotency and self-renewal, and by promoting non-neural differentiation once ESCs exit the pluripotent status. This explains why, in either mESCs or hESCs, both Activin/Nodal and BMP activities need to be low for neural induction to occur, despite the different roles of these pathways in mESC and hESC pluripotency.

### Neural conversion of ESCs by culture in TGFβ-free media or in the presence of exogenous TGFβ inhibitors

While high levels of TGFβ signaling are incompatible with neural induction in ESCs, a question of interest is whether culture conditions based on TGFβ-free media are sufficient for neuralization or whether exogenous TGFβ antagonists are needed. In other words, the contribution of ESCs to the levels of TGFβ signals in the culture and the effects of these endogenous TGFβ levels on ESC neuralization have to be carefully evaluated.

mESCs cultured in serum-free media in either monolayer or floating aggregate conditions readily undergo neuralization, which is suppressed by exogenous BMP or Nodal factors [43–45]. Treatments with BMP or Nodal antagonists, however, can only slightly increase the efficiency of mESCs neuralization [43–45]. Thus, endogenous TGFβ signaling is not strong enough to prevent mESC from acquiring neural cell fates, possibly also because of the expression of endogenous TGFβ antagonists in differentiating mESCs [43].

The situation in hESCs is more complex. When hESCs are cultured as floating aggregates at least for an initial period of differentiation, neuroectoderm generally forms in serum-free media even without exogenous BMP antagonists [34, 46, 47]. Inhibitors of Activin/Nodal signaling can accelerate the process of neural induction in these conditions [48, 49]. In contrast, exogenous BMP inhibitors are essential to achieve neural induction in adherent conditions, since monolayer hESC cultures differentiated without BMP antagonists are refractory to neuralization [50] and differentiate to extra-embryonic fates [37, 39] or to non-neural ectoderm [42]. Published evidence differs as to whether inhibitors of Activin/Nodal signaling are necessary along with BMP antagonists to enhance neural induction in these conditions [50–52]. Several factors may contribute to these discrepancies. First, adherent and non-adherent conditions may differentially modulate expression of TGFβ ligands and antagonists in hESCs [39, 42, 47]. Second, differently from mESCs, hESCs are frequently cultured in the presence of complex substrates (such as Matrigel) or supplements (such as serum replacements or feeder cell-conditioned media) that can potentially modulate the levels of TGFβ signaling in the cultures. Third, intrinsic differences in the hESC lines that are used need to be taken into account [53]. Overall, the levels of endogenous Activin/Nodal and/or BMP signaling tend to be higher in differentiating hESCs compared to mESCs, thus making them less prone to neuralization in the absence of exogenous TGFβ inhibitors. Further efforts are needed to understand the impact of different culture conditions on TGFβ pathway activation in hESCs.

### Fibroblast growth factor (FGF) signaling plays complex, stage-dependent roles in ESC differentiation towards neuroectoderm

Several studies have suggested that neural induction is not simply a default pathway of pluripotent cells shielded from TGFβ signaling and that a permissive influence of FGF signaling is implicated [2, 3]. Evidence obtained in vertebrate model organisms, however, has been inconsistent [33, 54–59].

Research with pluripotent stem cells has shown that neural induction is a multistep process that is differently influenced by FGF signaling at different steps, leading to a unifying view of the role of FGF signaling in neuroectoderm specification. mESCs represent an ICM-like basal pluripotency state (or ground state) that is intrinsically self-maintaining if shielded from inductive differentiation stimuli exerted by autocrine FGF4 [10, 11]. In a minimal medium depleted of LIF and serum, and supplemented with inhibitors of glycogen synthase kinase 3β (GSK3β, a component of the Wnt signaling pathway) and the mitogen-activated protein kinase activated by FGF4 (MAPK, also known as ERK), mESCs propagate and maintain ground state pluripotency [60]. Instead, mESCs with reduced FGF signaling are refractory to both neural and non-neural differentiation [43, 61]. These results have been explained by assuming that FGF signaling is critical in the transition from ground state pluripotency to an epiblast-like pluripotent state (or primed pluripotency), corresponding to EpiSCs directly derived from post-implantation epiblast. EpiSCs maintain expression of the pluripotency factors Oct4, Nanog and Sox2, but they are primed for multilineage, including neural, differentiation [62–64].

The subsequent roles of FGF signaling in neural induction can be best modeled in EpiSCs or hESCs, representing a primed pluripotency state. In hESCs, Activin/Nodal and FGF signaling cooperate in promoting Nanog expression, maintaining pluripotency and restraining differentiation [65, 66]. Following inhibition of TGFβ signaling, hESCs more readily lose expression of pluripotency markers and upregulate expression of the early neural markers Otx2 and Pax6 when FGF signaling is also downregulated [67].

Inhibition of TGFβ and FGF signaling, however, appears to drive hESCs to an early neural state that is not yet committed to evolve into definitive neuroectoderm. FGF inhibition beyond the first 4 days of hESC neural differentiation hampers neuroectoderm formation [47, 68, 69] and diverts early neural progenitors towards differentiation into peripheral nervous system-like neurons, similar to those originating from derivatives of the neuroectoderm/epidermis border (placodes and neural crest) in vivo [67]. On the contrary, exogenous FGF delivery following an initial period of hESC neural differentiation favors stabilization of definitive neuroectoderm [52, 68], which may be hindered by increased BMP signaling [52].

Further issues remain to be addressed to clarify the roles of FGF signaling in neural induction. In particular, different FGFs may trigger distinct intracellular pathways [70], which might have different roles in neuroectoderm specification [64]. Moreover, FGF signaling has a crucial influence in neural patterning (see below), which may affect the expression of neural markers that become restricted to selected CNS regions (such as Otx2 and Pax6 [71]). Thus, the genetic networks regulated in ESCs by FGF signaling in the context of neural induction need further investigation.

**Fig. 1:**
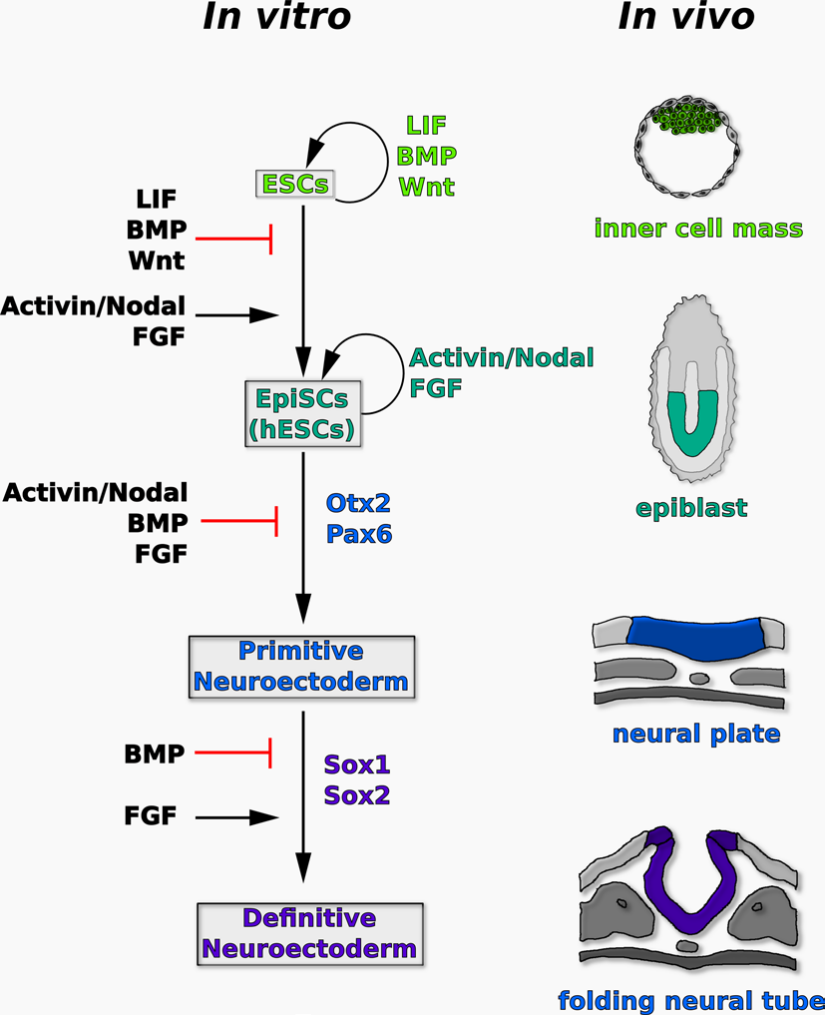
Proposed model of the signaling pathways controlling the transition of ESCs from ground state pluripotency to definitive neuroectoderm in vitro. Some of the key transcription factors regulated during these events are also indicated. Ground state ESCs self-renew in the presence of LIF, BMP, and Wnt/GSK3β-dependent signaling. They can be converted into primed epiblast-like cells (such as EpiSCs or hESCs) and maintained in this state under the influence of Activin/Nodal and FGF signaling. In these cells, downregulation of TGFβ and FGF signaling causes specification of early neural precursors expressing Otx2 and Pax6. Stabilization of these primitive precursors into Sox1/2-positive definitive neuroectoderm is inhibited by BMP signaling and supported by FGF signaling. The *drawings on the right* show the corresponding stages of mammalian development in vivo

A tentative model of neural induction in ESCs based on the work described in this section is shown in Fig. 1.

## Forebrain specification in neuralized ESCs: protecting neural progenitors from posteriorizing factors

In different experimental settings, once neural progenitors emerge from pluripotent cells, they almost invariably display AP positional identities, suggesting that allocation of AP fates is an integral part of neural induction or it is a very early process occurring in newly neuralized cells. Positional identities, however, are often initially plastic and can be modified by further exposure to patterning signals.

Embryological experiments in amphibians led to a two-step model of neural induction and AP patterning, known as the activation–transformation model [4]. In the activation step, the neural tissue is induced and at the same time acquires a forebrain identity. In the transformation step, part of the neuroectoderm is converted to posterior fates by caudalizing factors. According to this model, forebrain fates constitute a default positional identity in the early neuroectoderm that can be maintained by preventing exposure to posteriorizing signals.

### Downregulation of TGFβ signaling in ESCs leads to the specification of neuroectoderm with anterior positional identities

The activation–transformation model has been supported by studies performed in model organisms. In many assays of neural induction, whenever neural tissue is induced, it initially expresses forebrain-specific genes, suggesting a link between induction of neural identity and acquisition of an anterior character. This activation step has been commonly associated with the downregulation of TGFβ signaling in competent ectoderm. For example, impaired TGFβ signaling in frog ectoderm [72] or mouse epiblast [32] leads to induction of neuroectoderm expressing forebrain, but not hindbrain/spinal cord markers. Signals located in posterior parts of the embryo, including Wnts, FGF, and retinoic acid (RA), promote neuroectoderm posteriorization, thus acting as transformation signals, while antagonists of these signaling pathways are present in presumptive forebrain regions and protect them from posteriorization [4, 5, 17, 73, 74].

Work performed in ESCs has provided substantial confirmation for the activation–transformation model. In both mESCs and hESCs, protocols that allow spontaneous neural induction in the absence of exogenous signals lead to the generation of neural progenitors expressing markers of forebrain identity [46, 75–77]. As described above, depending on the experimental conditions used, hESCs often require exogenous TGFβ inhibitors for effective neural induction and these conditions too result in neuroectoderm with anterior positional character [51, 52, 78]. These observations strongly support the idea that neural development is initiated by an activation step that simultaneously imparts both neural and forebrain identities and which is triggered by decreased TGFβ signaling.

Among the earliest genes induced during neural conversion of hESCs are those coding for the Otx2 and Lhx2 transcription factors, which are selectively expressed in the early rostral neuroectoderm in vivo [6]. Both factors can directly bind enhancer regions of the *Pax6* gene and promote its expression in hESCs differentiating to neuroectoderm [67, 79]. Pax6 in turn acts as a critical determinant of hESC neuralization [80]. These studies suggest that neural conversion of ESCs is initiated by genes that also promote the specification of anterior positional fates, thus providing a direct molecular link between neuralization and the acquisition of anterior character during the activation step of neural induction.

### Roles of BMP and Activin/Nodal signaling in the AP patterning of ESC-derived neuroectoderm

Recent work in mESCs has revealed a novel function of BMP signaling in AP neural patterning distinct from its well-known role in neural induction [45]. This study showed that mESCs, when differentiated to neuroectoderm in minimal medium devoid of exogenous inducers, endogenously produced BMPs, which significantly affected the AP patterning of ESC-derived neuroectoderm. Control cultures acquired caudal forebrain and/or midbrain identities, which were shifted to rostral forebrain fates by inhibition of BMP signaling. BMPs were mainly produced by neuralized cells and BMP inhibitors affected the expression of AP markers only when added after ESC neuralization, indicating that the effects of BMP signaling on AP patterning were distinct from those on neural induction. These observations suggest that the rostral character shown by neuroectoderm formed in conditions of strong BMP inhibition may be due to suppression of both anti-neuralizing and posteriorizing BMP activities and raise the possibility that dynamic regulation of BMP signaling during early neural development in vivo is involved in the specification of different AP fates.

Work carried out in hESCs has shown that Activin/Nodal signaling too can affect both the induction of neuroectoderm and its AP patterning, since inhibition of Activin/Nodal signaling was found to impose a caudal positional identity on the induced neuroectoderm [49]. This is surprising due to the wide association of TGFβ pathway inhibition with anterior neural induction both in ESCs and in model organisms. Induction of posterior neuroectoderm following inhibition of Activin/Nodal signaling was observed in hESCs differentiated as floating aggregates [49]. In adherent hESCs cultures, instead, neural induction does not happen efficiently following single Activin/Nodal antagonism [37, 42, 50], while treatments with Activin/Nodal and BMP inhibitors cause anterior neural induction [51, 52, 78]. In hESC-derived floating aggregates, Activin/Nodal signaling has been shown to control differentiation of anterior visceral endoderm [34], a tissue which is implicated in AP neural patterning [17, 73, 74]. Thus, inhibition of Activin/Nodal signaling may cause neuroectoderm caudalization in floating aggregates by interfering with anterior visceral endoderm formation. In alternative, in adherent cultures, the posteriorizing effect of Activin/Nodal inhibition may be offset by the anteriorizing effect of simultaneously inhibiting the BMP pathway. Overall, it appears that both branches of TGFβ signaling may not solely control neuroectoderm formation but also its AP positional fates. More work, however, is needed to understand the mechanisms underlying the patterning effects of these pathways.

### Activation of Wnt/β-catenin, FGF, and RA signaling exert posteriorizing effects on ESC-derived neuroectoderm

As predicted by the activation–transformation model, forebrain neuroectoderm generated from ESCs by spontaneous neuralization or by means of exogenous TGFβ inhibitors can be caudalized to midbrain, hindbrain, and/or spinal cord fates by exposure to signaling molecules promoting posterior specification in vivo, such as Wnts, FGFs, or RA. For example, exogenous RA promotes spinal cord fates in neuroectoderm derived from mESCs or hESCs [50, 81]. Dose-dependent specification of midbrain or hindbrain/spinal cord fates has been demonstrated following experimental activation of Wnt/β-catenin signaling along with TGFβ inhibition in hESCs [82]. hESC neuralization in the presence of exogenous FGF2 has been shown to promote hindbrain/spinal cord fates [52], while FGF8 treatments have been used to facilitate specification of midbrain fates [50, 83]. Caudalizing effects of Wnt/β-catenin and FGF pathways have also been reported in mESCs [75, 84–86]. In these studies, downregulation of forebrain genes was detected along with upregulation of posterior neural markers. These caudalizing effects were observed both with treatments started from early stages of ESC differentiation, or with staggered treatments started after an initial period of neuralization, when expression of anterior neural markers became detectable in differentiating ESCs [46, 50, 75]. Thus, studies performed in ESCs show that posterior specification can be temporally separated from neural induction and it can occur in progenitors pre-induced towards anterior neuroectoderm.

Studies in ESCs are starting to provide interesting insights on how different caudalizing pathways interact during the specification of posterior neural fates. For example, in hESC-derived neuroectoderm, exogenous Wnt antagonists were shown to reduce the posteriorizing effects of FGF2 treatments [52], indicating that the FGF and Wnt/β-catenin pathways collaborate in posterior neural specification. In another study, hESCs neuralized by TGFβ inhibition initially expressed forebrain markers, but shifted to midbrain expression profiles following withdrawal of TGFβ inhibitors and exposure to exogenous FGF2 [87]. This posteriorization was due to downregulation of the Wnt inhibitor Sfrp1 and enhancement of Wnt signaling after the switch from TGFβ inhibition to FGF2 treatments, suggesting that FGF signaling may influence AP neural patterning indirectly by facilitating Wnt pathway activation [87]. Exogenous FGF2, however, was able to hamper forebrain specification and increase posterior gene expression in hESC-derived neuroectoderm even in the presence of Wnt antagonists [52], suggesting that FGF signaling may also exert Wnt-independent posteriorizing effects.

The crucial influence of the Wnt/β-catenin pathway in the AP patterning of ESC-derived neuroectoderm has been confirmed by enhanced forebrain specification following abrogation of endogenous Wnt/β-catenin during ESC neuralization. Recent reports have described activation of endogenous Wnt/β-catenin signaling during neural conversion of hESCs by TGFβ inhibition [52, 88]. As shown by treatments with Wnt/β-catenin inhibitors, this endogenous Wnt/β-catenin activation restrained induction of rostral forebrain fates in favor of caudal forebrain and/or midbrain fates [52, 88]. Other studies, in which neuralized hESCs predominantly acquired rostral forebrain fates, reported upregulation of endogenous Wnt antagonists during neural induction [77, 89]. Similar results have been reported in mESCs [44]. Activation of the β-catenin-dependent branch of the Wnt pathway causes nuclear accumulation of β-catenin, which then promotes transcription of Wnt target genes together with TCF and LEF factors [90]. Wnt pathway inhibitors acting at the level of β-catenin were more effective than extracellular Wnt antagonists in repressing specification of caudal forebrain and/or midbrain fates [52, 88], suggesting that, depending on the culture conditions, Wnt-independent activation of β-catenin signaling may contribute to neuroectoderm posteriorization. Another study described increased expression of rostral forebrain markers and reduced expression of caudal forebrain and/or midbrain markers following neuralization of mESCs and hESCs in the presence of PI3K/AKT pathway inhibitors, or of mESCs in insulin-free media [76]. This suggests that insulin, a common additive in serum-free protocols, may be the source of Wnt-independent β-catenin activation in ESC cultures working via upregulation of the PI3K/AKT pathway. Regulation of β-catenin via PI3K/AKT signaling, however, appears to be complex and context-dependent [91, 92]. Hence, more work is needed to elucidate the sources of Wnt-independent β-catenin signaling during ESC differentiation.

Consistently with the posteriorizing effects of exogenous FGF during ESC neuralization, inhibition of both FGF and TGFβ signaling in hESCs can facilitate the upregulation of early forebrain markers, such as *Otx2*, *Lhx2* and *Foxg1*, at early stages of neural conversion [78]. While the impact of endogenous FGFs on the AP patterning of ESC-derived neuroectoderm should be further investigated, this study suggests that functional FGF signaling is not required for the initial specification of forebrain fates in ESCs.

**Fig. 2:**
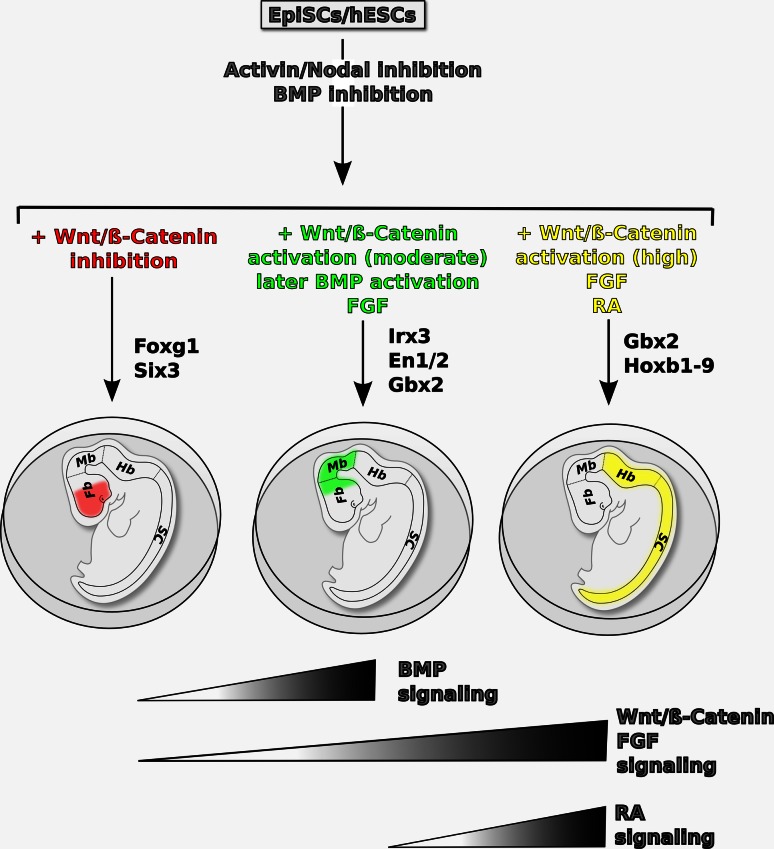
Proposed model of the signaling pathways controlling AP patterning in pluripotent stem cell-derived neural progenitors in vitro. Neural progenitors induced by inhibition of TGFβ signaling in primed pluripotent cells (EpiSCs or hESCs) can be steered to distinct AP fates, marked by specific transcription factors, in different culture conditions. Inhibition of both TGFβ and Wnt/β-catenin signaling allows specification of rostral forebrain fates, marked by FoxG1 and Six3 expression. Activation of BMP, Wnt/β-catenin and FGF signaling with appropriate timing and doses promotes caudal forebrain and/or midbrain fates, marked by Irx3, En1/2, and Gbx2 expression. Posteriorization to hindbrain/spinal cord fates (expressing Gbx2 and Hoxb1-9) can be achieved by high levels of Wnt/β-catenin, FGF2, and/or RA signaling. The *drawings* show the main AP subdivisions of the developing mammalian nervous system in vivo. *Fb* forebrain, *Mb* midbrain, *Hb* hindbrain, *sc* spinal cord

A model of AP neural patterning in pluripotent stem cells is schematized in Fig. 2. This model illustrates how it is possible to direct differentiation of pluripotent stem cells towards region-specific neural fates using protocols that recapitulate the mechanisms of AP neural patterning described in vertebrate model organisms.

## Patterning discrete forebrain identities: default or instructive signaling-driven differentiation?

According to the prosomeric model [93], the forebrain can be subdivided into a rostral region (or secondary prosencephalon) and a caudal region (or diencephalon). The rostral forebrain includes the telencephalon dorsally, the hypothalamus ventrally and the optic vesicles evaginating from the lateral walls of the hypothalamus. The caudal forebrain contains, from rostral to caudal, the prethalamus, the thalamus, and the pretectum. While the caudal forebrain lies posterior to the rostral forebrain both at the neural plate and neural tube stages [5], the topological relationships of the telencephalon, the eye field, and the hypothalamus are very dynamic and differ in the neural plate compared to the neural tube. Fate mapping, gene expression, and in vivo time-lapse analyses in different vertebrates have shown that, at the neural plate stage, the presumptive telencephalon is found in the most anterior neuroectoderm rim, with the eye field behind it and the hypothalamic progenitors occupying medial/caudal positions with respect to the eye field [5, 94, 95]. Each of these structures becomes regionalized along the dorsoventral axis. For example, the telencephalon is subdivided into the pallium (or cortical telencephalon) and the subpallium (including the medial and lateral ganglionic eminences, or MGE and LGE) [7], the eye field into presumptive retina and optic stalk [8], and the hypothalamus is also patterned into dorsal and ventral hypothalamic regions [93]. A central issue that is not addressed by the activation–transformation model is how this variety of anterior regional fates is specified by the simple default mechanism associated with the activation step.

Specification of caudal forebrain fates represents a mild form of posteriorization of the rostral neuroectoderm induced with the activation step. For example, moderate levels of BMP and/or Wnt signaling can cause specification of caudal forebrain and/or midbrain fates in neuralized ESCs at the expense of rostral forebrain fates [45, 52, 82, 88]. These observations suggest that the caudal forebrain arise from the transformation, rather than the activation step of neural induction. However, how does the activation step lead to the specification of telencephalic, ocular, and hypothalamic fates?

### Downregulation of extracellular signaling in ESCs allows a default telencephalic specification pathway

Chemically defined protocols of ESC neuralization represent an excellent system to investigate the intrinsic and extrinsic mechanisms governing the specification of different rostral forebrain fates. When mESCs were differentiated as floating aggregates in medium devoid of any undefined supplement and exogenous signaling factors, including insulin, they acquired rostral hypothalamic fates [76]. In these non-adherent conditions, specification of telencephalic or retinal cell fates required more complex media that included TGFβ and Wnt antagonists and/or undefined reagents such as serum replacement (KSR), Matrigel, or serum [44, 96–98]. These observations suggest that rostral hypothalamic fates may represent the default specification pathway of pluripotent stem cells in the absence of exogenous patterning signals. Yet, absence of exogenous inducers does not equate with an intrinsic specification mechanism, since endogenous extracellular signals are present in differentiating ESC cultures and they may play a role in rostral forebrain patterning.

In support of this idea, when mESCs were cultured in adherent conditions using a minimal medium devoid of exogenous morphogens and a low cell density to minimize endogenous extracellular signaling, they were efficiently specified to telencephalic progenitors [99]. Telencephalic specification was also achieved in mESCs or hESCs cultured in minimal media containing TGFβ inhibitors or both TGFβ and Wnt/β-catenin inhibitors [45, 88]. In addition, rapid upregulation of telencephalic genes was detected in hESCs treated with both TGFβ and ERK pathway inhibitors [78]. Based on these studies, it is tempting to speculate that telencephalic specification is the true default fate of pluripotent stem cells when the antineuralizing and posteriorizing activities of endogenous TGFβ, Wnt/β-catenin, and possibly ERK signaling, are repressed in the absence of exogenous patterning signals. This is an attractive hypothesis due to the rostral-most position occupied by the telencephalic primordium in the early vertebrate neural plate [5]. Validation of this default mechanism, however, will need a formal demonstration that these more stringent culture conditions, minimizing both exogenous and endogenous extracellular signals, are selectively permissive for the specification of telencephalic, but not hypothalamic fates. KSR has been shown to promote telencephalic fates in mESCs [44, 97], and it has been included in some protocols of telencephalic conversion in hESCs [97], raising the possibility that telencephalic specification may not be entirely independent from extrinsic signals. Lysophosphatidic acid contained in KSR has been shown to influence the response of hESCs to Wnt factors [100], but whether and how this mechanism is implicated in the patterning of ESC-derived neuroectoderm has not been currently addressed.

### DV patterning of ESC-derived telencephalic progenitors depends on dose-dependent and stage-dependent regulation of Wnt/β-catenin and hedgehog signaling

Several studies have investigated whether ESC-derived telencephalic progenitors acquire dorsal (pallial) or ventral (subpallial) character in culture. mESCs or hESCs differentiated to telencephalon in conditions allowing expression of the endogenous Sonic hedgehog (Shh) signaling molecule, or including exogenous agonists of the Shh pathway, acquired ventral identities [88, 99, 101–103]. These results are consistent with the established role of Shh as a key ventralizing morphogen during neural patterning in vivo [104]. Instead, culture conditions where Shh signaling remained quiescent, or was inhibited by exogenous antagonists, generated progenitors with dorsal telencephalic identities [45, 97, 99, 101, 105], which upon terminal differentiation gave rise to cortical neurons. A distinctive feature of cortical development in vivo is that laminar fate and subtype specification are linked to neuron birth-date, with early born neurons settling in deep cortical layers and late-born neurons populating the upper layers [106]. Outstandingly, similar temporal patterns have been observed in ESC-derived cortical progenitors, which can generate different neuronal subtypes following neurogenic waves comparable to those described in vivo [97, 99, 105]. In three-dimensional culture, cortical cells obtained from mESCs or hESCs formed self-organized structures containing different cell zones arranged along the apico-basal axis and resembling the in vivo cortical layers [97]. These observations suggest that, when cultured in permissive conditions devoid of antineuralizing, posteriorizing, and ventralizing signals, pluripotent stem cells undergo a default cortical differentiation pathway giving rise to cortical progenitors, which are able to generate a lineage of distinct neuronal subtypes in vitro following the same timing schedule of cortical progenitors in vivo.

Studies performed in hESCs have shown that endogenous Wnt/β-catenin signaling can play an instructive role in the specification of dorsal telencephalic fates in vitro [88, 101], which is consistent with the role played by this pathway during pallial development in vivo [107]. Expression of endogenous Wnt ligands was detected in culture conditions promoting spontaneous differentiation of hESCs to dorsal telencephalon [101]. When Wnt/β-catenin signaling was inhibited during the acquisition of DV identities, ventral telencephalic markers were moderately upregulated if the Shh pathway was left intact [88]. Furthermore, Wnt/β-catenin inhibition strongly sensitized hESC-derived telencephalic progenitors to the ventralizing effects of exogenous agonists of Shh signaling [88, 101]. Conversely, treatments with exogenous Wnt ligands during an appropriate temporal window enhanced expression of dorsal telencephalic genes [101]. Similar results have also been reported following manipulation of the Wnt/β-catenin pathway in telencephalic progenitors obtained from mESCs [44]. In these studies, treatments with Wnt/β-catenin inhibitors did not abrogate the expression of dorsal telencephalic markers in the absence of exogenous Shh agonists [88, 101], suggesting that default cortical specification of ESCs can still take place, albeit less efficiently, when Wnt/β-catenin is inactive.

**Fig. 3:**
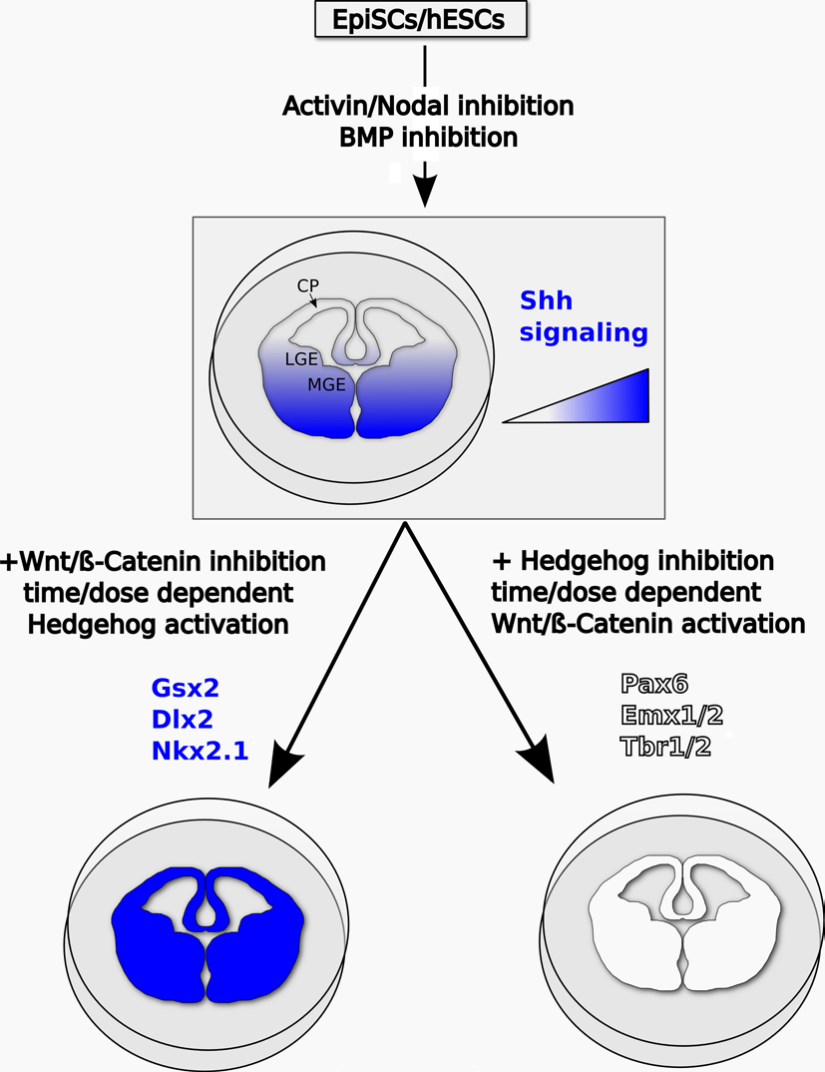
Proposed model of the signaling pathways controlling DV patterning in pluripotent stem cell-derived telencephalic progenitors in vitro. Telencephalic character can be induced in primed pluripotent stem cells by antagonism of TGFβ signaling and transient inhibition of Wnt/β-catenin. Protracted Wnt/β-catenin inhibition, together with dose-dependent and time-dependent Shh pathway activation, can steer telencephalic progenitors to LGE and/or MGE fates, marked by Gsx2, Dlx2, and Nkx2.1 expression. Dorsal telencephalic identities, associated to expression of Pax6, Emx1/2, and Tbr1/2, can be imposed by inhibition of Shh signaling and controlled activation of Wnt/β-catenin signaling. The *drawings* show the main dorsoventral subdivisions of the developing mammalian telencephalon in vivo. *CP* cortical pallium, *LGE* lateral ganglionic eminence, *MGE* medial ganglionic eminence

In conclusion, it appears that, in ESC-derived telencephalic progenitors, active Shh signaling is necessary for ventral patterning, while active Wnt/β-catenin signaling can enhance, but it is not absolutely required for, dorsal specification. The model in Fig. 3 shows how the combined manipulation of these signaling pathways can be used to efficiently drive neuralized ESCs to ventral or dorsal telencephalic fates.

### Eye field specification in ESCs involves inductive extracellular cues

During embryonic development, the region of the anterior neural plate comprised between the telencephalon and the diencephalon becomes specified as the eye field, which contains the progenitors fated to give rise to the retina and the adjoining optic stalk [5, 71, 108]. In contrast to telencephalic fates, the generation of retinal progenitors from ESCs appears to depend on both endogenous and exogenous inductive signals and tends to be suppressed when extracellular signaling is minimized. This suggests that retinal conversion is not the result of a simple default mechanism but relies on extrinsic mechanisms of cell fate specification.

hESCs can acquire telencephalic fates when cultured in minimal media containing inhibitors of TGFβ and Wnt/β-catenin pathways [88, 103]. These conditions, however, may not be compatible with robust expression of eye field genes. In a recent study, adherent hESCs allowed to differentiate in the presence of Wnt/β-catenin inhibitors but not TGFβ inhibitors showed a marked increase in eye field gene expression levels compared to cultures treated with TGFβ inhibitors, even though hESC neuralization was less efficient [52]. Treatments with different combinations of inhibitors suggested that both Activin/Nodal and BMP inhibition concurred in repressing transcription of eye field genes. In timing experiments, Activin/Nodal and BMP inhibitors mainly affected eye-field gene expression during specific temporal windows [52]. Therefore, constitutive inhibition of Activin/Nodal and BMP signaling throughout the stages of neural induction and patterning may not be permissive for efficient eye field specification, which may require fine manipulation of the timing and levels of activation of these pathways.

Other studies have shown that hESCs can be converted to retinal progenitors by means of protocols employing floating aggregate culture conditions, which facilitate cell–cell interactions, and avoiding exogenous morphogens or inhibitors, thus leaving endogenous extracellular signaling intact [77, 109]. In these studies, about 20 % of the aggregates upregulated expression of retina-specific genes and generated eye-like structures containing retinal cells, while the remaining aggregates acquired telencephalic fates. These observations suggest that three-dimensional cultures relying on endogenous signaling pathways were permissive for eye field specification and retinal development in hESCs, but additional instructive cues were required to efficiently drive forebrain progenitors towards retinal fates and away from telencephalic fates.

A few exogenous molecules or supplements increasing retinal conversion of ESCs have been described [108]. Delivery of Activin/Nodal factors can promote the generation of retinal progenitors from mESCs [96, 98], while IGF1 [110, 111], Shh agonists [112], nicotinamide [113], and N2/B27 supplements [111, 114] can enhance retinal conversion of hESCs, but their mechanisms of action in eye field formation, both in vitro and in vivo, are poorly understood. Most of these studies used media supplemented with KSR to facilitate eye field specification. The effects of KSR were dose-dependent, since either KSR omission or high KSR levels were not permissive for retinal cell formation in mESCs [98], while induction of retinal fates in hESCs required high KSR levels [112]. The specific KSR components involved in these effects are not currently known.

Unexpectedly, the most important influence on retinal conversion of mouse or human ESCs has been shown to be exerted by Matrigel (BD Biosciences, Franklin Lakes, NJ, USA), a reconstituted basement membrane preparation. Culture of mESCs as floating aggregates in the presence of low KSR and Matrigel improved the yield of eye field progenitors from 15 % to up to 70 % compared to previous studies, with up to 80 % of the aggregates positives for retinal markers [98, 115]. Importantly, Matrigel stimulated both retinal fate specification and morphogenesis, since ESC-derived retinal progenitors gave rise to eye-like structures that produced different retinal neuron populations arranged in a proper apico-basal order [98, 112, 115]. Two recent reports have described fast, efficient generation of retinal progenitors in hESCs cultured as cell clumps embedded in Matrigel with a minimal medium supplemented only with N2 and B27 [111, 116]. This approach resulted in striking neural tube-like neuroepithelial structures with nearly uniform expression of eye field markers after just 5 days and high numbers of photoreceptor cells after further culture in specific differentiation conditions [111, 116]. It should be noted, however, that Matrigel includes both extracellular matrix components and soluble factors. While some studies described efficient generation of retinal cells using a growth factor-reduced version of Matrigel [115], others showed that growth factors present in Matrigel, such as IGF1, contribute to its retinal-promoting activity [111]. Notably, the effects of Matrigel could be mimicked by a combination of defined matrix proteins (purified laminin and entactin) and the Nodal signaling factor [98]. More work will be needed to clarify the mechanisms of Matrigel-dependent retinal conversion of ESCs, including the impact of extracellular matrix on the production and/or activity of endogenous growth factors and morphogens.

## A model of early forebrain induction and patterning

In summary, studies performed in ESCs allow to draw a model of early forebrain induction and patterning in pluripotent progenitors, which provides a useful framework for future investigations on forebrain development both with ESCs in vitro and with model organisms in vivo.

According to this model, downregulation of TGFβ and FGF signaling initiates neural lineage specification in primed epiblast-like progenitors (such as EpiSCs or hESCs), while stabilization of this primitive neuroectodermal state into definitive neuroectoderm requires FGF signaling and is hampered by increased BMP signaling (Fig. 1).

Inhibition of TGFβ signaling in primed pluripotent cells (EpiSCs or hESCs) induces a loosely defined anterior neuroectoderm population (possibly including both rostral and caudal forebrain fates as well as midbrain fates). On this ground, inhibition of Wnt/β-catenin signaling allows efficient specification of rostral forebrain fates. Moderate levels of BMP, Wnt/β-catenin, and FGF signaling during specific time windows promote caudal forebrain and/or midbrain fates at the expense of rostral forebrain fates. High levels of Wnt/β-catenin, FGF2, and/or RA signaling cause strong neuroectoderm posteriorization to hindbrain/spinal cord fates (Fig. 2).

Telencephalic specification appears to be the default rostral forebrain fate induced in primed pluripotent cells by downregulation of TGFβ signaling and transient inhibition of Wnt/β-catenin, and possibly of ERK signaling. DV patterning of these telencephalic progenitors is dependent on the levels and timing of activation of the Shh and Wnt/β-catenin pathways, with Shh signaling steering telencephalic progenitors to ventral (LGE and/or MGE) fates and Wnt/β-catenin signaling promoting dorsal telencephalic identities (Fig. 3). Specification of alternative rostral forebrain fates, such as eye field, seems to depend on extrinsic inductive cues, which remain only partially understood.

## Conclusions and perspectives

In this review, we have attempted a systematic scrutiny of the existing literature in the field of anterior neural development in ESCs, with a focus on the molecular mechanisms of early cell fate decisions. For this reason, our discussion has been limited to the early steps of forebrain development and it has not included, or only briefly mentioned, later steps of neuronal cell differentiation and maturation. A detailed description of the mechanism of forebrain development in vivo was beyond the scope of this review and it has been extensively covered elsewhere [5, 6, 73, 74]. Nonetheless, it is important to note how the fundamental mechanisms of early neural development appear to be conserved between vertebrate embryos in vivo and ESCs in vitro. For example, genetic studies in frog and fish embryos pointed to the importance of TGFβ inhibition in neural induction. Thus, depletion of multiple TGFβ ligands in these systems causes expansion of the neuroectoderm at the expense of non-neural ectoderm, while depletion of organizer-derived TGFβ antagonists has the opposite effect [18–21]. These approaches have been difficult to replicate in amniote embryos, but work in ESCs has confirmed that TGFβ inhibition is a universal requirement in neural induction. Some of the findings in mouse mutant embryos, however, appear to contradict work in ESCs. For example, abrogation of the BMP and Nodal antagonist Cerberus-like does not prevent neural induction in mice, as expected from ESC work [117]. Such discrepancies may be explained by the fact that multiple antagonists contribute to the regulation of TGFβ signaling in vivo and their simultaneous tissue-specific and stage-specific inactivation is challenging. In fact, studies on mouse mutants for TGFβ ligands or their receptors have been supportive of work in ESCs [32, 33]. Furthermore, studies on both vertebrate embryos in vivo and on ESCs in vitro have lent support to the activation–transformation model of neural induction and AP patterning as the one best explaining data obtained in each system. In both cases, anterior forebrain appears to be the default positional fate of the neuroectoderm induced by TGFβ inhibition and it can be posteriorized by Wnt, FGF and/or RA signaling, or stabilized if protected from these caudalizing signals. Crucially, ESCs have allowed to study these events with higher resolution than that usually achieved in vivo and in particular to define the minimal extrinsic signaling and the time of competence required to specify pluripotent progenitors towards distinct neural identities, as well as the intermediate steps occurring between the pluripotent stage and the committed neural progenitor stage. Future investigations will need to clarify to what extent the refined models of neural induction and patterning emerging from ESC work are faithful to the mechanisms operating in vivo.

Pioneering studies performed in ESCs have also greatly advanced our understanding of how complex anatomical structures are formed during development and opened new avenues of investigation in this field. It will be of great interest to work out whether forebrain morphogenesis from ESCs in vitro recapitulates the same mechanisms used by embryonic cells to form forebrain structures in vivo, or whether similar morphological structures can be formed using alternative, context-dependent routes. Last but not least, the tremendous progress of recent ESC research in neural development has represented a significant step forward in the in vitro generation of neural tissue for clinical applications and has paved the way to the next generation of studies for stem cell-based therapies.
